# Global Mapping of Telemedicine Regulation and Ethical Safeguards: Mixed Methods Exploratory Document Analysis

**DOI:** 10.2196/86613

**Published:** 2026-04-22

**Authors:** Rosamaria Rodrigues Gomes, Carlos Adriano Silva dos Santos, Ivone Duarte, Rui Nunes

**Affiliations:** 1 Faculty of Medicine, Universidade do Porto, Alameda Prof. Hernâni Monteiro, Porto, 4200-319, Portugal, 55 82996216868; 2 Medical School, Centro Universitário CESMAC, Maceio, Brazil

**Keywords:** telemedicine, health equity, digital health, informed consent, privacy, bioethics

## Abstract

**Background:**

Telemedicine has become central to digital health strategies, yet the regulatory environment that shapes ethical safeguards and equitable access remains uneven and incompletely assessed across countries. Legal and normative instruments matter because they define requirements for privacy, consent, accountability, professional readiness, and barrier reduction.

**Objective:**

This study aimed to map the current global landscape of normative instruments related to telemedicine and identify which ethical and social safeguards are explicitly addressed, with particular attention to equity.

**Methods:**

We conducted a document analysis guided by the READ (ready the materials, extract data, analyze data, and distill findings) framework. From February 2024 to February 2026, we conducted a structured web-based search across all World Health Organization (WHO) member states with no language restrictions, using official government sources, statutory professional regulators, and institutional publication channels. Retrieval combined internal site searches, direct navigation, external search engine queries, and targeted snowball sampling to identify currently in-force instruments. Two researchers independently extracted and coded data using a predefined codebook. We operationalized 10 binary items covering regulatory presence and scope (questions 1 and 2), safeguards for data protection (question 3), consent and disclosure (questions 4 and 5), prior in-person prerequisites (question 6), monitoring (question 7), training requirements (question 8), and equity (questions 9 and 10). We summarized frequencies overall and stratified by WHO region and World Bank income group and conducted a qualitative thematic analysis of included normative instruments.

**Results:**

Of the 194 WHO member states, 81 (41.8%) had at least one current normative instrument related to telemedicine in force. Among these, 72.8% (59/81) defined telemedicine or telehealth. Data protection provisions were most common (73/81, 90.1%), followed by mandatory informed consent (n=71, 87.7%) and monitoring mechanisms (n=65, 80.2%). Fewer countries required disclosure of telemedicine limitations (n=36, 44.4%) or mandated telemedicine-specific training (n=26, 32.1%). Prior in-person consultation requirements were uncommon (n=8, 9.9%). Equity-related safeguards were uneven: 51.9% (n=42) referenced justice, equity, or nondiscrimination, whereas 30.9% (n=25) included concrete barrier reduction provisions (eg, digital inclusion or accommodations for people with disabilities and minors).

**Conclusions:**

Telemedicine regulation is becoming more common, but both coverage and safeguarding content remain uneven. While privacy, consent, and monitoring are frequently addressed where regulation exists, disclosure, physician competency, and operational equity measures are less consistently specified. Strengthening telemedicine governance will require translating ethical commitments into enforceable standards that address digital determinants of access and protect groups at risk of exclusion.

## Introduction

Telemedicine is part of the World Health Organization’s (WHO) global strategy on digital health for 2020 to 2025, which aims to improve access to health services [1] and achieve the goal of universal health care [2]. The expansion of telemedicine after the COVID-19 pandemic was not accompanied by universal health coverage, which stagnated between 2019 and 2021 [3].

One criterion for measuring telemedicine progress is the assessment of established legal frameworks [4]. Notably, the legal context for telemedicine activities has not been sufficiently assessed [5]. Previously, a 2016 WHO report stated that 67% of countries had no legislation or policy to define jurisdiction over eHealth actions [2]. Conceptually, telemedicine is commonly situated within telehealth, which, in turn, is part of broader digital health or eHealth. However, both scholarship and policy documents acknowledge that these labels are inconsistently used across settings, with eHealth often treated as an umbrella term that includes telehealth and telemedicine, among other subdomains [6]. In addition, definitions vary widely, with over 100 definitions recognized in the literature [7]. For the purposes of this study, “telemedicine” refers to the provision of clinical care or clinical support at a distance using information and communications technologies [4,8]. To maximize cross-country comparability when legal instruments used different labels, we treated “telemedicine” and “telehealth” as interchangeable terms for remote clinical care mediated by information and communications technologies.

Regulation is especially relevant because telemedicine depends on governance arrangements that define safeguards for privacy, consent, accountability, and equitable access. Recent work emphasizes that data governance regulations have often lagged digital health innovation and that many low- and middle-income countries (LMICs) lack digital data governance strategies [9]. This gap can undermine patient trust as concerns about privacy, security, and downstream uses of data may reduce willingness to share data and mishandling or reidentification can lead to stigmatizing harms in low-resource settings [9].

When dividing countries by income group, the statistics show a clear difference: 55% of the high-income group vs 5% of the low-income group report having defined policies or legislation addressing eHealth [2]. This characterization by income group is essential because the adoption of telemedicine services aims to improve health outcomes for the most disadvantaged populations, particularly in LMICs [10]. At the same time, evidence syntheses focused on low- and lower-middle–income contexts describe persistent constraints related to weak governance and the lack of legal and policy frameworks, as well as the role of poor or vague eHealth strategies as barriers to sustainable implementation [6].

Therefore, this study addressed the following research question: what is the current state of telemedicine legislation worldwide? Our study aimed to provide a global overview of the existing legal frameworks regarding telemedicine and identify the ethical and social concerns associated with implementing and practicing telemedicine within current norms, particularly in terms of equity.

## Methods

### Overview

We conducted a document analysis and followed the READ framework, which includes 4 steps: ready the materials, extract data, analyze data, and distill findings [11]. The unit of analysis was the country. Our objective was to map, for each WHO member state, whether any current telemedicine-related normative instrument existed and which ethical safeguards were explicitly addressed in those instruments.

### Search Strategy and Data Sources

We performed a structured web-based document search from February 2024 to February 2026 with no language restrictions. Documents in English, Portuguese, Spanish, French, and Italian were reviewed in the original language. For other languages, machine translation (Google Translate) was used to translate documents into English for eligibility assessment and data extraction. For each WHO member state, we searched telemedicine-related normative instruments through three complementary routes: (1) official government sources, prioritizing ministries of health and other health authorities, national gazettes, and legislative repositories; (2) professional regulatory bodies such as medical councils or boards for binding rules, codes, or official guidance regulating telemedicine practice; and (3) institutional publication channels that compile or discuss telemedicine regulation, including reports from the WHO, International Bar Association [12], Inter-American Development Bank (IDB) [8], and the Organisation for Economic Co-operation and Development [13].

Core search terms included “telemedicine,” “digital health,” and “e-health” adapted to each jurisdiction’s terminology. We also captured “telehealth” when it appeared in the title, text, indexing, or cross-referenced materials of retrieved instruments and in jurisdictions where “telehealth” was the locally preferred term. However, “telehealth” was not used as a uniform stand-alone core search term across all country searches. Retrieval used internal website search tools when available, direct navigation to legislation or regulation sections, and external search engine queries combining the country name and/or issuing authority with the core terms to identify documents not easily retrievable through site navigation. We also used targeted snowball sampling by following references within retrieved documents (eg, amendments, implementing decrees, and cross-referenced policies) to identify the most current and authoritative versions. Our primary focus was on national-level instruments applicable to clinical telemedicine practice; when subnational instruments (as in federations where regulation is enacted at the state or provincial level) were encountered, they were recorded but not used to define the presence of country-level regulation unless they were from the highest applicable authority in that jurisdiction.

### Eligibility Criteria and Exclusions

We included normative instruments that (1) were issued by a government authority or a statutory regulatory body (including medical councils or boards), (2) explicitly addressed the provision of telemedicine or telehealth, and (3) were current and in force at the time of extraction. We considered regulation primarily as “a rule or order issued by an executive authority or regulatory agency of a government and having the force of law” [14]. However, we also included binding rules, codes, or official guidance issued by governments or medical boards when they contained normative requirements for telemedicine practice. We excluded nonofficial commentary; media reports; secondary analyses; documents that were not current or authoritative; and instruments that were explicitly labeled as drafts or in a planning phase, including proposed bills and planning stage strategies. When multiple versions existed, we retained the most recent version in force and documented the substituted instruments as background.

### Constructing the Global Overview

We produced a global overview covering WHO member states and WHO regions classified by income level according to the World Bank [15]. WHO membership includes 194 countries divided into 6 regions. World Bank income groups are stratified as high income, upper-middle income, lower-middle income, and low income, with 1 country, Venezuela, listed as unclassified in the 2024 list. Niue and the Cook Islands are WHO member countries not included in the World Bank classification. For country-level mapping, the presence of any eligible in-force telemedicine-related instrument was coded as “regulatory mechanism present.” The absence of an identified eligible document was recorded as “no instrument identified,” which should not be interpreted as evidence that telemedicine activities do not occur in that setting.

### Data Extraction and Development of Extraction Questions

In step 2 of the READ approach, we extracted data into spreadsheets organized by WHO region. Telemedicine was selected as the analytic focus because the extraction domains were anchored in the IDB framework for telemedicine regulation [8], which provides a structured, previously developed set of regulatory dimensions that can be operationalized consistently across countries.

The set of questions (Textbox 1) was defined by mapping the IDB framework maturity model [8] domains across their 7 questionnaires and adapting it to 2 analytic aims: first, to capture the presence and basic scope of telemedicine regulation (questions 1 and 2) and, second, to capture core ethical safeguards commonly addressed in telemedicine governance, including privacy and data protection (question 3) when present within telemedicine-related instruments (or when those instruments explicitly incorporated general privacy rules); informed consent and disclosure of the limits of telemedicine (questions 4 and 5); and an ethically dual-use prerequisite with implications for both continuity and access (question 6), that is, whether norms mandate a prior in-person consultation before telemedicine is permitted, as well as monitoring mechanisms (question 7) and professional competence requirements (question 8). Equity was treated as a core safeguard domain operationalized through 2 complementary items: question 9 captured whether norms explicitly referenced justice, equity, or nondiscrimination as a guiding principle, and question 10 captured whether norms included concrete barrier reduction provisions such as digital inclusion measures, accessibility accommodations for persons with disabilities, or specific safeguards for minors. Each question was operationalized as a binary variable with predefined criteria for what constituted an explicit normative statement. Full operational definitions and decision rules are provided in Multimedia Appendix 1. We piloted the extraction form on a purposive subset of countries across different WHO regions and income levels to refine definitions, clarify ambiguous items, and harmonize coding decisions before full extraction.

Textbox 1. **Data extraction questions.**

- Question 1: are there regulatory aspects in telemedicine?
- Question 2: do the norms define telemedicine?
- Question 3: are there concerns about the protection of personal data?
- Question 4: is informed consent mandatory?
- Question 5: do the norms require that patients be provided with information about the limits of telemedicine?
- Question 6: do the norms require prior in-person consultation?
- Question 7: is there a monitoring mechanism in place?
- Question 8: is it necessary to have any training in telemedicine to provide care in this modality?
- Question 9: do the norms mention the principle of justice in any way?
- Question 10: is there a guarantee for reducing access barriers (digital inclusion or accommodations for minors and people with disabilities)?

For each country, we downloaded all documents included in the study, and for each of the 10 questions, responses were coded as binary (“yes” or “no”) and recorded in a Microsoft Excel spreadsheet available in the Figshare repository (Digital Science; see the Data Availability section). “Yes” required an explicit normative statement addressing the item. In instances in which a country possessed multiple eligible instruments, the item was designated as “yes” if any currently in-force instrument explicitly included a provision addressing the item. Ambiguous cases were deliberated and resolved through consensus.

### Data Quality Assurance

All eligible documents were independently reviewed and coded by 2 researchers using the predefined codebook. For countries with multiple eligible instruments, coding decisions were based on review of all in-force documents identified for that jurisdiction. Discrepancies were resolved in consensus meetings with reference to the underlying document text and the operational definitions in the codebook. We also conducted peer debriefing to review the coherence of country-level coding and the interpretation of cross-country patterns.

### Quantitative and Qualitative Analysis

In step 3 of the READ approach, we analyzed data quantitatively using descriptive statistics. We calculated counts and proportions of countries meeting each item in Textbox 1 overall and stratified by WHO region and World Bank income group.

In parallel, we conducted a qualitative contextual review of the extracted text excerpts and relevant sections of the normative instruments. All eligible in-force instruments identified in countries with a regulatory mechanism present were included in this qualitative review. We read the full text of each included document and extracted passages relevant to the analytic domains. Qualitative analysis was primarily deductive, using the extraction domains to organize and compare material across jurisdictions. We then conducted a secondary interpretive pass to identify recurring regulatory framings, omissions, and equity-related concerns that were not fully captured by the binary items. These observations were discussed within the team and used to contextualize the quantitative patterns, with particular attention to ethical and social implications and equity-related safeguards. In step 4 of the READ approach, we distilled findings by integrating quantitative results and qualitative observations into a narrative synthesis of global regulatory coverage and gaps.

### Ethical Considerations

Although this study did not constitute human subject research because it was limited to document analysis of publicly available sources, it was submitted to the Ethics Committee of the Faculty of Medicine of the University of Porto for confirmation and received administrative approval under the number 115/CEFMUP/2023. Informed consent was not applicable.

## Results

### Regulatory Concentration and Basic Scope (Questions 1 and 2)

Across the 194 WHO member states, 81 (41.8%) countries had at least one current telemedicine normative instrument in force (question 1). Of these 81 countries, 59 (72.8%) included an explicit definition of telemedicine or telehealth in the regulatory text (question 2). Table 1 provides a condensed overview of all items; fully stratified results for each question are provided in Multimedia Appendix 2.

**Table 1.** Condensed overview of telemedicine regulatory coverage and safeguards.

Domain and measure	Countries, n/N (%)
Regulatory presence and scope	
Any current in-force telemedicine-related normative instrument (question 1)	81/194 (41.8)
Explicit definition of telemedicine or telehealth (question 2)	59/81 (72.8)
Data protection	
Privacy or data protection provision (question 3)	73/81 (90.1)
Consent and disclosure	
Mandatory informed consent (question 4)	71/81 (87.7)
Disclosure of telemedicine limitations (question 5)	36/81 (44.4)
Access conditions	
Prior in-person consultation required (question 6)	8/81 (9.9)
Oversight	
Monitoring or oversight mechanism (question 7)	65/81 (80.2)
Professional readiness	
Telemedicine-specific training requirement (question 8)	26/81 (32.1)
Equity principles	
Justice, equity, or nondiscrimination referenced (question 9)	42/81 (51.9)
Barrier reduction	
Provision to reduce access barriers (question 10)	25/81 (30.9)

### Data Protection (Question 3)

Protection of personal data was the most frequently addressed safeguard. Data protection or privacy-related provisions were identified in 90.1% (73/81) of the countries.

### Informed Consent and Disclosure of the Limits of Telemedicine (Questions 4 and 5)

Mandatory informed consent for telemedicine was required in 87.7% (71/81) of the countries (question 4). In contrast, fewer than half (36/81, 44.4%) required that patients be informed about the limitations of telemedicine (question 5).

### Prior In-Person Consultation (Question 6)

Only 9.9% (8/81) of the countries mandated a prior in-person consultation before telemedicine was permitted.

### Monitoring and Professional Competence Requirements (Questions 7 and 8)

Monitoring or oversight mechanisms relevant to telemedicine were present in 80.2% (65/81) of the countries (question 7). Specific telemedicine training requirements for providers were less common, appearing in 32.1% (26/81) of the countries (question 8).

### Equity-Related Safeguards (Questions 9 and 10)

Equity was unevenly addressed. In total, 51.9% (42/81) of the countries referenced justice, equity, or nondiscrimination as a guiding principle within telemedicine norms (question 9). However, only 30.9% (25/81) of the countries included explicit provisions for reducing barriers, such as digital inclusion measures or accommodations for people with disabilities and minors (question 10).

## Discussion

Despite the global increase in telemedicine regulations, a significant regulatory divide persists. High-income regions have established robust norms, whereas low-income countries lack specific frameworks, particularly for data protection, informed consent, professional accountability, and equity.

### Regulatory Concentration and Global Inequity

Telemedicine regulation remains unevenly distributed worldwide, with substantial variation across regions and income groups. In our updated mapping, 81 countries had identifiable telemedicine regulations.

Compared with earlier WHO survey findings on telemedicine and eHealth [2], which reported that 33% of countries had some form of norms in this area, our mapping identified in-force telemedicine instruments in 41.8% (81/194) of WHO member states. Because the WHO survey relied on self-reported responses and used broader eHealth categories, these estimates are not directly comparable; however, the difference suggests that formal regulatory instruments are now identified in a larger share of countries than previously reported.

A central ethical implication is that the presence of telemedicine norms does not necessarily translate into improving access to health care [5]. When legal standards are absent, incomplete, or poorly specified, key ethical and equity-oriented safeguards become fragmented and are often delegated to institutional policies, private platforms, or professional discretion [16-18]. This governance structure can distribute protections and accountability unevenly across settings.

These dynamics matter because telemedicine is frequently promoted as a tool to expand access and advance universal health coverage [1-3,10]. It tends to benefit populations with greater connectivity, health literacy, and institutional coverage while leaving socioeconomically vulnerable groups with fewer protections and fewer pathways for redress when harm occurs [19-21].

If regulation is developed primarily in areas with already strong health care access, telemedicine may disproportionately benefit populations with greater connectivity, literacy, and institutional coverage unless safeguards for equity are operationalized [1-3,10]. Regulation, in this sense, becomes part of the distributive architecture of health systems, shaping who receives protection, under what standards, and with which mechanisms of accountability.

### Data Protection and the Commodification of Health Data

The findings show that data protection was widely recognized in telemedicine norms, with most regulated countries addressing privacy and confidentiality. This is ethically significant because telemedicine intensifies data flows across platforms, providers, and institutions, increasing the risks of unauthorized access, secondary use, and informational harms [18,19,22,23]. In many jurisdictions, data protection requirements may be anchored in general frameworks such as the General Data Protection Regulation [24] or HIPAA (Health Insurance Portability and Accountability Act) [25], but the operational implications for telemedicine still depend on how specific telemedicine rules define responsibilities, data governance, and accountability mechanisms [18].

The Americas and Europe still lead in privacy protection, as the 2016 WHO report [2] indicates, which aligns with our findings. Despite 90.1% (73/81) of the countries demonstrating concern about data protection in their norms, only 3.7% (3/81) were low-income countries. However, privacy provisions by themselves do not ensure equity. As health data are increasingly treated as a commodity, governance structures must address power asymmetries in data collection and use [18].

A privacy clause can coexist with limited patient understanding, weak enforcement capacity, and platform-driven practices that disproportionately burden low-resource settings [18,19,22,23]. For regulations oriented toward equity, privacy protections should be paired with clear obligations regarding transparency, security standards, data sharing, and remedies for misuse, particularly when patients have limited bargaining power and few alternatives.

### Informed Consent, Disclosure, and Telemedicine-Specific Understanding

A substantial proportion of telemedicine frameworks (71/81, 87.7%) required informed consent, indicating broad recognition that remote care entails clinical and informational risks [18,19,22,23]. However, consent requirements are ethically robust only when they are paired with adequate disclosure of telemedicine limitations. Among the 71 countries with consent requirements, 60.6% (43/71) were in Europe and the Americas versus 39.4% (28/71) elsewhere, and 95.8% (68/71) were in higher-income settings versus 4.2% (3/71) in low-income settings.

Although consent was highly valued in the norms, the obligation to address modality-specific constraints was not explicit, so it risks becoming a procedural formality rather than a meaningful safeguard [19,22]. Specific telemedicine consent should communicate what can and cannot be safely managed remotely; when escalation to in-person care is required; and how patient data are stored, shared, and protected [19,26].

Disclosure obligations are also equity relevant. Individuals may consent without meaningful alternatives, adequate understanding of technical limitations, or the connectivity needed to use telemedicine effectively [19,21-23]. When telemedicine is adopted as the default pathway, the ethical risk is a 2-tiered standard of explanation and care in which disadvantaged populations receive weaker disclosure and weaker protection [18]. Therefore, strengthening telemedicine-specific disclosure can protect autonomy while also reducing preventable harm among populations with lower health and digital literacy.

### Prior In-Person Consultation and the Balancing of Equity

Requirements for prior in-person consultation illustrate how regulation can create equity concerns. While an initial face-to-face encounter may strengthen clinical accuracy and the therapeutic relationship [18,27], mandating in-person contact before telemedicine can create barriers for geographically isolated or economically constrained populations and may worsen inequities [28]. Although only 9.9% (8/81) of the norms mandated prior in-person consultation, 5 of those were from LMICs, which often face practical and clinical barriers.

A more equity-sensitive approach is to define clinical and risk-based criteria for when in-person evaluation is necessary while preserving patient choice and ensuring safe escalation pathways [28]. Such criteria-based regulation can support safety without embedding avoidable access barriers into law. It also aligns legal requirements with clinical realities, recognizing that appropriate use varies by condition, setting, and patient needs.

### Monitoring and Professional Competence

As telemedicine expands, regulatory attention often turns to oversight and fraud prevention [29,30]. Monitoring mechanisms were present in 80.2% (65/81) of the identified frameworks, although oversight criteria were often described in broad terms [29,31]. Europe and the Americas accounted for 48.1% (39/81) of the countries with a monitoring mechanism in place, whereas only 3.7% (3/81) of low-income countries had one. There is consensus that monitoring can deter malpractice and protect populations, but given ongoing debates about telehealth fraud, oversight should be transparent and proportionate [29,31].

In parallel, training requirements are an important marker of professional readiness. Remote care requires specific competencies in communication, risk triage, documentation, privacy practices, and clinical escalation [5,7,22,32,33]. Among the analyzed norms, 67.9% (55/81) did not require any telemedicine training regardless of country region. All low-income countries in the sample fell within this “no training requirement” group. Where training is not expected, practice may become more variable, and clinicians may compensate for uncertainty through overtesting or overprescribing, increasing costs and potentially undermining quality [7,32,33]. Therefore, training standards can operate as both quality and equity safeguards by reducing avoidable variation across providers and settings and by supporting safer telemedicine use where specialist support is limited.

Since the WHO first documented it, there has been concern about the need for robust governance in the monitoring and evaluation of telemedicine, transforming the digital encounter from a private transaction into a transparent, auditable clinical act that protects both patient safety and the physician’s professional integrity [2,4].

### From “Equity Language” to Enforceable Duties

The global overview indicated that justice is increasingly referenced in telemedicine norms (42/81, 51.9%). However, ethical language does not automatically translate into enforceable protections [19-21,34,35]. This gap matters because many determinants of effective telemedicine use are not clinical. They are conversion factors and digital determinants of health that ascertain whether formal availability translates into genuine access. This includes aspects such as connectivity, affordability, literacy, accessible design, language support, disability accommodations, and social protection [19,21,34,36]. The capabilities approach by Sen [37] helps clarify why access to technology alone does not guarantee substantive freedom to obtain care, particularly for disadvantaged groups whose autonomy and dignity are constrained.

In LMICs, socioeconomic conditions pose challenges to implementing telemedicine, and existing norms often fail to operationalize equitable access. Among the norms studied, justice was cited in only 2.4% (1/42) of those from low-income countries compared with 40.5% (17/42) of those from high-income countries. Higher-income patients often use telemedicine more than underserved populations [27], raising ethical concerns about prioritizing coverage for advantaged groups.

The presence of barrier reduction guarantees in a meaningful share of countries (25/81, 30.9%) is an important advance, but it also highlights whether equity is treated as an aspirational principle or as a compliance requirement [19-21,34,35]. Without enforceable duties, telemedicine may expand primarily among those already digitally connected, which conflicts with the “leave no one behind” principle [35].

This also creates a risk of equity washing; references to justice that are not linked to enforceable obligations can remain rhetorical rather than protective [38]. Through a capabilities and digital determinant lens, equity-oriented regulation requires duties that translate equity-relevant conversion factors into minimum standards and accountability expectations rather than relying solely on general commitments to justice [19-21,34].

### Limitations and Implications

This study has limitations inherent to global document analysis. First, it depends on the availability and retrievability of official documents through web-based sources and may underrepresent jurisdictions where relevant norms exist but are not publicly accessible online. Second, written legal and regulatory texts do not necessarily reflect implementation, enforcement capacity, resource constraints, or routine practice. Third, definitional heterogeneity persists across jurisdictions; although we used an inclusive approach and triangulated sources, variation in what counts as “telemedicine regulation” remains. Fourth, our search strategy was adapted to each jurisdiction and captured “telehealth”-labeled instruments when identified through local terminology, indexing, or cross-references, but “telehealth” was not used as a uniform stand-alone core search term across all country searches. Accordingly, some instruments indexed exclusively under telehealth may have been missed. Fifth, our corpus focused on formal normative instruments (eg, laws, regulations, codes, and official guidance) and did not systematically include other sources that may shape governance in practice, such as case law, litigation trends, administrative decisions, contractual arrangements, or platform-level rules.

These limitations are important for interpreting the findings. The results are best understood as a map of the documented normative landscape rather than a direct measure of implementation or regulatory effectiveness. Future research should examine how regulatory provisions are implemented and enforced and whether they are associated with reduced barriers in practice. Additional work is also needed on how jurisdictions allocate rights and responsibilities over data generated through telemedicine, including data subject rights, secondary uses, and cross-border data transfers. Consumer-facing digital health use (eg, online health information seeking and conversational artificial intelligence tools) was outside the scope of this study and warrants separate analysis because it raises distinct regulatory questions.

### Conclusions

Telemedicine regulation is present in a substantial share of WHO member states. However, significant gaps remain across countries in both coverage and content. Our mapping shows that telemedicine normative instruments are concentrated in higher-income settings and that key safeguards are implemented unevenly across jurisdictions, including data protection, informed consent, disclosure of telemedicine limitations, oversight mechanisms, provider competence requirements, and measures intended to reduce access barriers. These findings mainly impact governance. Legal texts indicate formal regulatory priorities but do not capture implementation, enforcement capacity, or outcomes. Nevertheless, identifying which safeguards are consistently addressed and which are frequently absent helps clarify where minimum protections are likely weak and where equity provisions remain underdeveloped. Future research should extend beyond cataloging laws to assess implementation and structural effectiveness, including whether regulatory choices translate into accountability, patient protection, and more equitable access in practice.

## Supplementary material

10.2196/86613 Multimedia Appendix 1 Codebook for telemedicine regulation mapping.

10.2196/86613 Multimedia Appendix 2 Stratified results and graphs.

## Abbreviations

HIPAA: Health Insurance Portability and Accountability Act
IDB: Inter-American Development Bank
LMIC: low- and middle-income country
WHO: World Health Organization
