# Supplementary material for: Global Mapping of Telemedicine Regulation and Ethical Safeguards: Mixed Methods Exploratory Document Analysis
Source: JMIR Form Res. 2026 Apr 22;10:e86613. doi: 10.2196/86613 (PMC13102328; doi:10.2196/86613)
Supplement: Multimedia Appendix 1 [file formative-v10-e86613-s001.pdf]

Codebook for Telemedicine Regulation Mapping (Q1–Q10)

General coding rules (apply to all questions)

- **Unit of coding:** country.
- **Documents eligible for coding:** current and in force normative instruments issued by government authorities or statutory professional regulators (laws, decrees, ministerial ordinances, regulations, binding board rules, official guidance with normative requirements).
- **Evidence standard for "Yes":** an **explicit normative statement** (must/shall/required/prohibited/authorized subject to conditions).
- **Country level "Yes":** code **Yes if any** eligible instrument in force contains qualifying language.
- **Ambiguity rule:** if the instrument uses aspirational language only (encourage/should consider) **without** imposing a requirement, code **No**
- **For each Question 1 "Yes":** download the document

QUESTION	INFORMATION	YES	NO
**Q1. Are there regulatory aspects in telemedicine?**	**W**hether the country has any **normative instrument** that regulates telemedicine practice.	At least one current, authoritative instrument that **authorizes, restricts, defines, or sets conditions** for telemedicine/telehealth services.<br>• legal authorization of telemedicine<br>• regulatory requirements for providing telemedicine<br>• licensing/registration rules specific to telemedicine	• only policy statements, speeches, non official reports<br>• only "digital health strategy" without operational norms and explicitly stated as planning stage or draft<br>• evidence only of telemedicine projects without normative instrument

		• prescribed standards of practice for telemedicine encounters	
**Q2. Do the norms define telemedicine?**	The instrument provides a **definition** of telemedicine or telehealth, typically specifying remote provision of clinical services using ICT.	• a section stating “telemedicine means…” or equivalent;<br>• a definitional clause that distinguishes telemedicine from telehealth, teleconsultation, telediagnosis, telemonitoring, etc.	• term is used without definition
**Q3. Are there concerns about the protection of personal data?**	The telemedicine instrument **requires or explicitly references** privacy, confidentiality, data protection, security measures, retention, data sharing limits, or compliance with a data protection law.	• explicit duty to protect privacy/confidentiality in telemedicine<br>• security requirements (encryption, authentication, secure platforms)<br>• prohibition of unauthorized recording/sharing<br>• compliance clause (for example “in accordance with the data protection act”) tied to telemedicine	• No data handling requirements

**Q4. Is informed consent mandatory?**	The instrument explicitly requires **patient consent** for telemedicine or for specific telemedicine acts (consultation, recording, data transmission).	• "informed consent is required" for telemedicine; or<br>• requirement to inform the patient and obtain consent before remote care; or<br>• explicit opt in or documented agreement	• only general consent for treatment is referenced without mention of telemedicine<br>• patient "agreement" is implied but not required
**Q5. Do the norms require that patients be provided with information about the limits of telemedicine?**	Explicit duty to inform patients about **limitations or risks** of telemedicine (diagnostic limits, inability to examine, technology failures, privacy risks) or to provide information allowing an informed choice between modalities.	• required disclosure of limitations/risks of remote modality<br>• requirement to inform about when in person assessment is needed<br>• requirement to inform about alternatives, including in person care	• only general patient information duties are stated<br>• "consent" is required but no mention of limitations, risks, or alternatives
**Q6. Do the norms require prior in person consultation?**	The instrument requires an in-person first visit before telemedicine is permitted, or restricts telemedicine to follow-up care after an in-person evaluation.	• explicit statement that initial consultation must be face to face<br>• telemedicine permitted only for follow up, established patient, or continuity of care	• telemedicine allowed for first contact with clinical discretion<br>• instrument encourages, but does not mandate, in person for first visit

**Q7. Is there a monitoring mechanism in place?**	the instrument establishes any formal mechanism for **oversight, monitoring, audit, reporting, accreditation, inspection, licensing, or enforcement** specifically relevant to telemedicine services or platforms.	• requirement to register telemedicine providers/platforms<br>• authority assigned to oversee telemedicine compliance<br>• audit or reporting obligations<br>• complaint mechanisms specific to telemedicine<br>• sanctions for non compliance tied to telemedicine requirements	• only general malpractice discipline exists with no telemedicine linkage<br>• "monitoring is encouraged" without specifying body or process
**Q8. Is it necessary to have any training in telemedicine to provide care in this modality?**	The instrument requires training, certification, competency assessment, or continuing education specific to telemedicine (technical, clinical, privacy, and platform use).	• explicit requirement to complete training prior to providing telemedicine<br>• certification or credentialing for telemedicine<br>• mandatory competency standards for telemedicine delivery	• training is recommended but not required<br>• general medical licensing/CPD with no telemedicine specificity
**Q9. Do the norms mention the principle of justice in any way?**	The instrument explicitly mentions justice, equity, non-discrimination, universal access,	• explicit term (justice, equity, equitable access,	• general health system values are mentioned in a

	priority to underserved groups, or fairness in access **as a normative principle** tied to telemedicine.	non discrimination) in the telemedicine context<br>• requirement to ensure access for underserved populations or to prevent exclusion	preamble with no telemedicine linkage<br>• equity is discussed only descriptively or aspirationally without normative framing
**Q10. Is there a guarantee for reducing access barriers (digital inclusion, accommodations for minors and people with disabilities)?**	The instrument includes explicit, actionable provisions to reduce barriers to telemedicine access for vulnerable groups, such as disability accommodations, language accessibility, minors' protections, affordable access, connectivity support, assisted telemedicine points, or alternative modalities.	• accessibility requirements (for example compliance with accessibility standards, assistive technologies)<br>• accommodations for disabilities or sensory impairments<br>• rules for minors, guardianship, assent, and child specific safeguards<br>• requirements for inclusive design or support channels for low digital literacy<br>• obligations to provide access points or support for underserved areas	• general equity statements without concrete measures<br>• statements like “should consider digital inclusion” without obligations
