# Supplementary material for: Global Mapping of Telemedicine Regulation and Ethical Safeguards: Mixed Methods Exploratory Document Analysis
Source: JMIR Form Res. 2026 Apr 22;10:e86613. doi: 10.2196/86613 (PMC13102328; doi:10.2196/86613)

AM = AMERICAS	SE ASIA = SOUTHEAS ASIA	EM = EASTERN MEDITERRANEAN
EU = EUROPE	WP = WESTERN PACIFIC	AF = AFRICA

ARE THERE REGULATORY ASPECTS IN TELEMEDICINE?

	AM	EU	SE ASIA	WP	EM	AF	WORLD	PROPORTION
HIGH	6	24	0	5	4	0	39	48%
LOW INCOME	0	0	0	0	0	3	3	4%
UPPER MIDDLE	13	4	3	2	0	1	23	28%
LOWER MIDDLE	1	2	4	3	4	1	15	19%
UNCLASSIFIED	1	0	0	0	0	0	1	1%
TOTAL	21	30	7	10	8	5	81	100%

	TOTAL	PROPORTION
WHO MEMBERS	194	100%
COUNTRIES YES TO TELEMED NORMS	81	42%

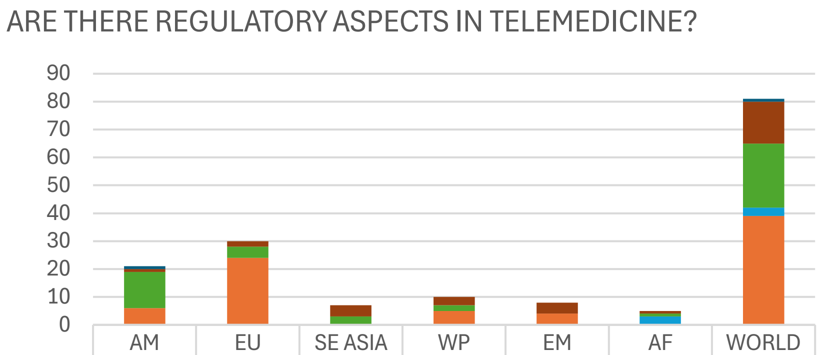

	AM	EU	SE ASIA	WP	EM	AF	WORLD
UNCLASSIFIED	1	0	0	0	0	0	1
LOWER MIDDLE	1	2	4	3	4	1	15
UPPER MIDDLE	13	4	3	2	0	1	23
LOW INCOME	0	0	0	0	0	3	3
HIGH	6	24	0	5	4	0	39

DO THE NORMS DEFINE TELEMEDICINE?

	AM	EU	SE ASIA	WP	EM	AF	WORLD	PROPORTION		
HIGH	5	13	0	5	4	0	27	33%	YES	73%
LOW INCOME	0	0	0	0	0	2	2	2%	NO	27%
UPPER MIDDLE	11	3	3	2	0	1	20	25%		
LOWER MIDDLE	0	0	4	2	2	1	9	11%		
UNCLASSIFIED	1	0	0	0	0	0	1	1%		
TOTAL	17	16	7	9	6	4	59	73%		

BY INCOME					BY REGION					
HIGH	LOW INC	UPPER MI	LOWER M	UNCLASS	AM	EU	SE ASIA	WP	EM	AF
27	2	20	9	17	17	16	7	6	6	4

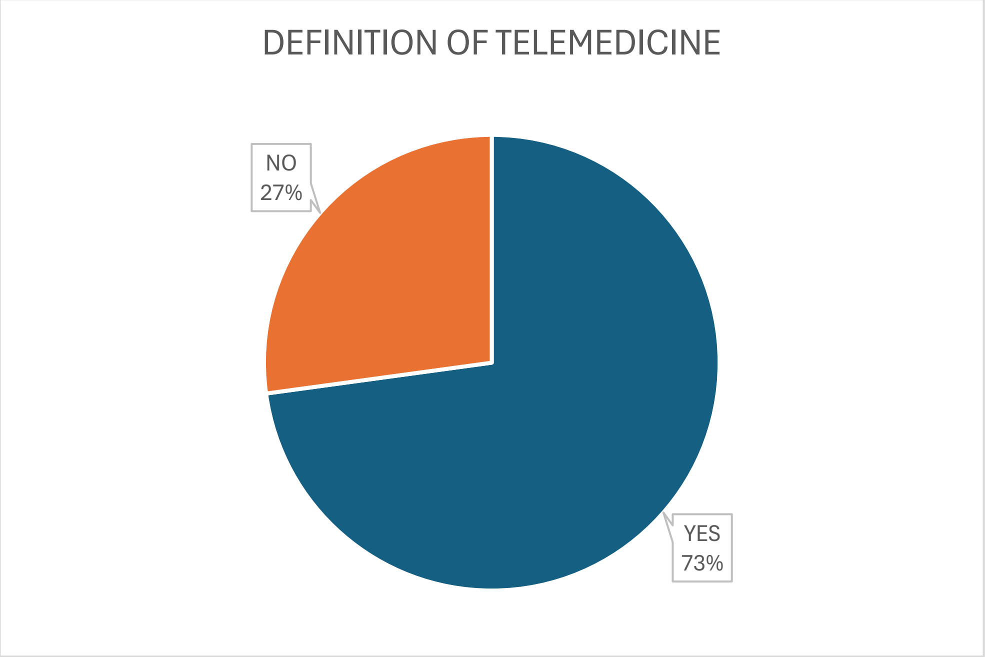

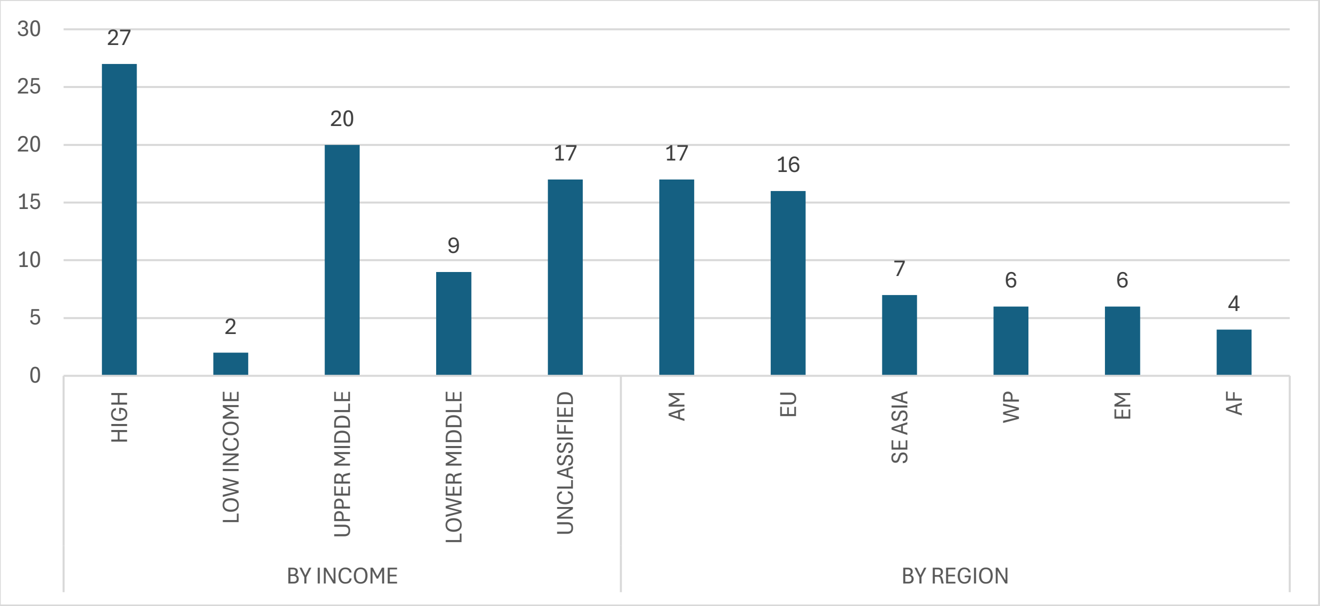

ARE THERE CONCERNS ABOUT THE PROTECTION OF PERSONAL DATA?

	AM	EU	SE ASIA	WP	EM	AF	WORLD	PROPORTION		
HIGH	6	19	0	5	4	0	34	42%	YES	90%
LOW INCOME	0	0	0	0	0	3	3	4%	NO	10%
UPPER MIDDLE	13	4	3	2	0	1	23	28%		
LOWER MIDDLE	1	0	4	2	4	1	12	15%		
UNCLASSIFIED	1	0	0	0	0	0	1	1%		
TOTAL	21	23	7	9	8	5	73	90%		

BY INCOME					BY REGION					
HIGH	LOW INC	UPPER MI	LOWER M	UNCLASS	AM	EU	SE ASIA	WP	EM	AF
34	3	23	12	1	21	23	7	9	8	5

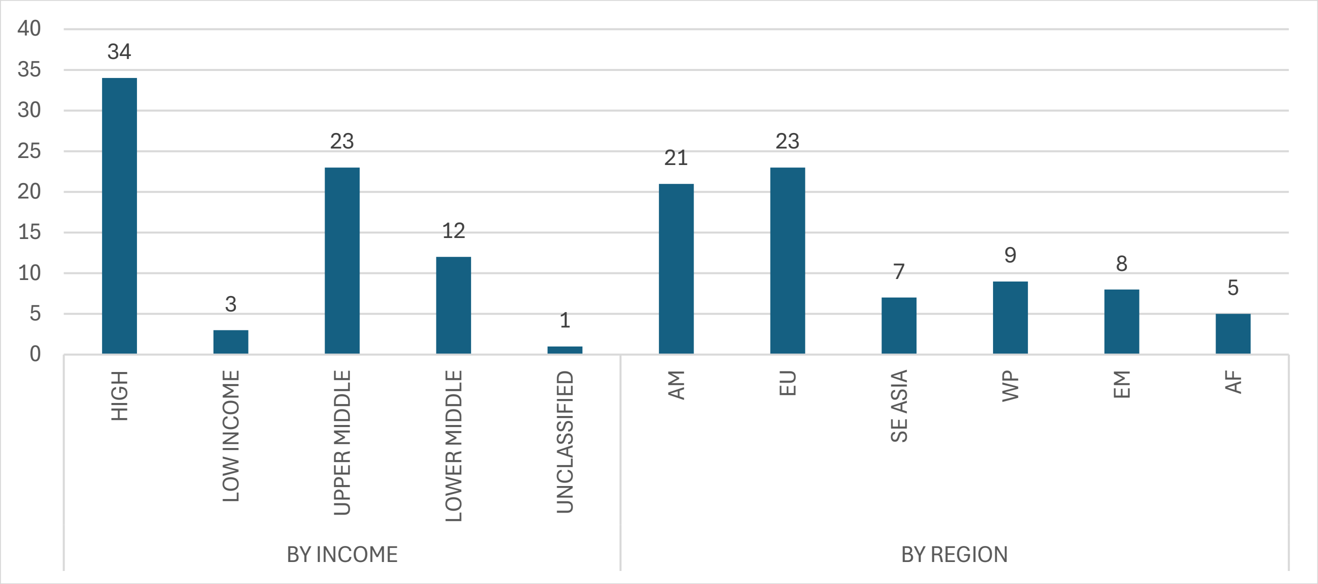

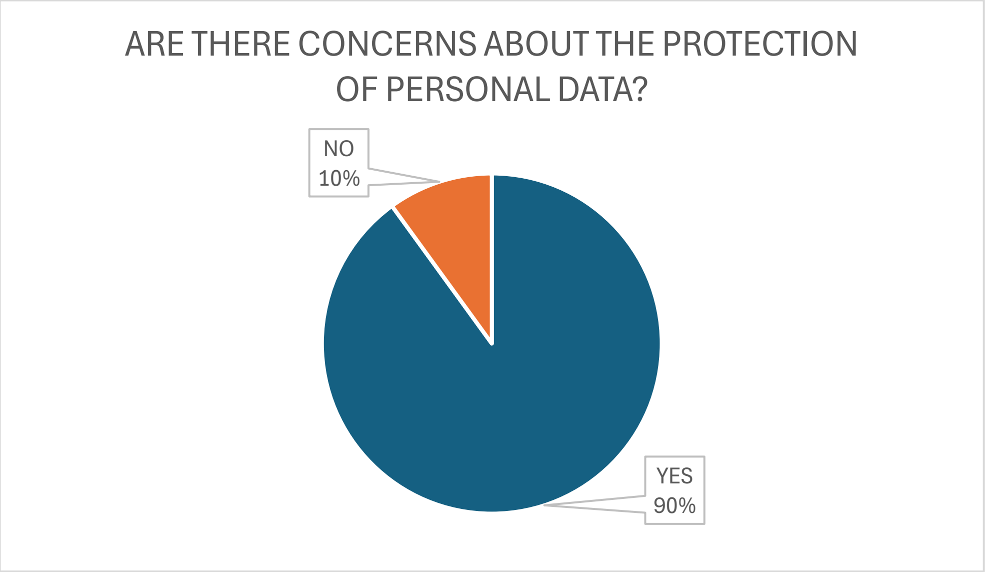

IS INFORMED CONSENT MANDATORY?

	AM	EU	SE ASIA	WP	EM	AF	WORLD	PROPORTION		
HIGH	5	21	0	5	4	0	35	43%	YES	88%
LOW INCOME	0	0	0	0	0	3	3	4%	NO	12%
UPPER MIDDLE	12	4	3	2	0	1	22	27%		
LOWER MIDDLE	0	0	4	2	3	1	10	12%		
UNCLASSIFIED	1	0	0	0	0	0	1	1%		
TOTAL	18	25	7	9	7	5	71	88%		

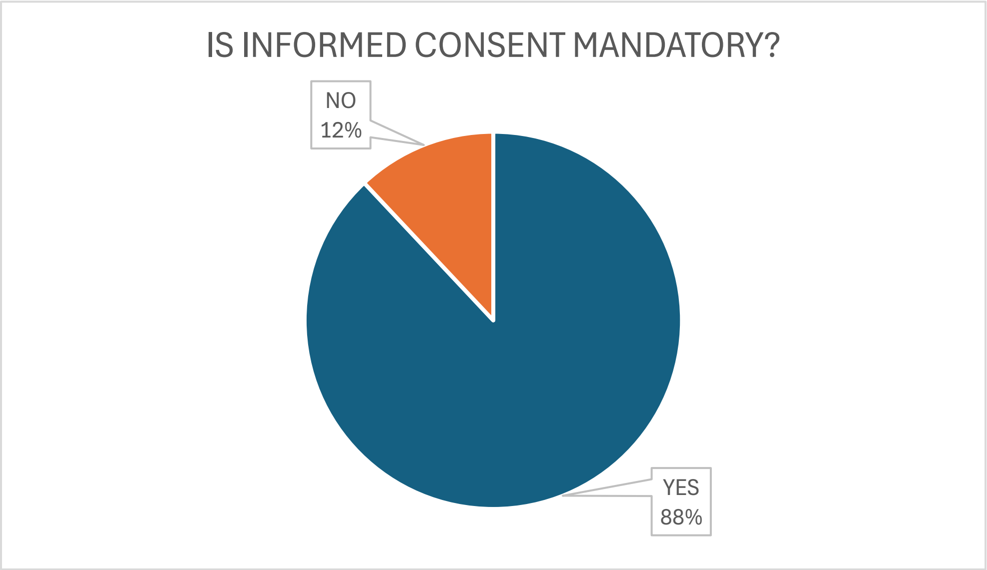

BY INCOME					BY REGION					
HIGH	LOW INC	UPPER MI	LOWER M	UNCLASS	AM	EU	SE ASIA	WP	EM	AF
35	3	22	10	1	18	25	7	9	7	5

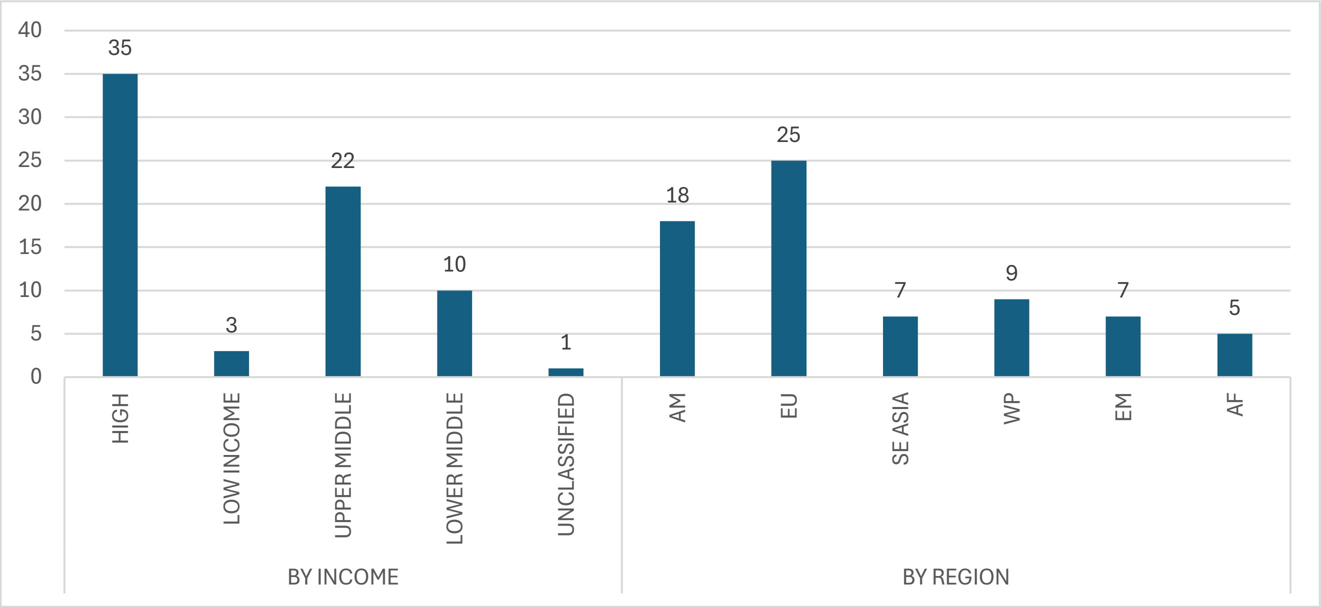

DO THE NORMS REQUIRE THAT PATIENTS BE PROVIDED WITH INFORMATION ABOUT THE LIMITS OF TELEMEDICINE?

	AM	EU	SE ASIA	WP	EM	AF	WORLD	PROPORTION		
HIGH	4	7	0	2	3	0	16	20%	YES	44%
LOW INCOME	0	0	0	0	0	1	1	1%	NO	56%
UPPER MIDDLE	8	1	2	2	0	0	13	16%		
LOWER MIDDLE	0	0	4	1	1	0	6	7%		
UNCLASSIFIED	0	0	0	0	0	0	0	0%		
TOTAL	12	8	6	5	4	1	36	44%		

BY INCOME					BY REGION					
HIGH	LOW INC	UPPER MI	LOWER M	UNCLASS	AM	EU	SE ASIA	WP	EM	AF
16	1	13	6	0	12	8	6	5	4	1

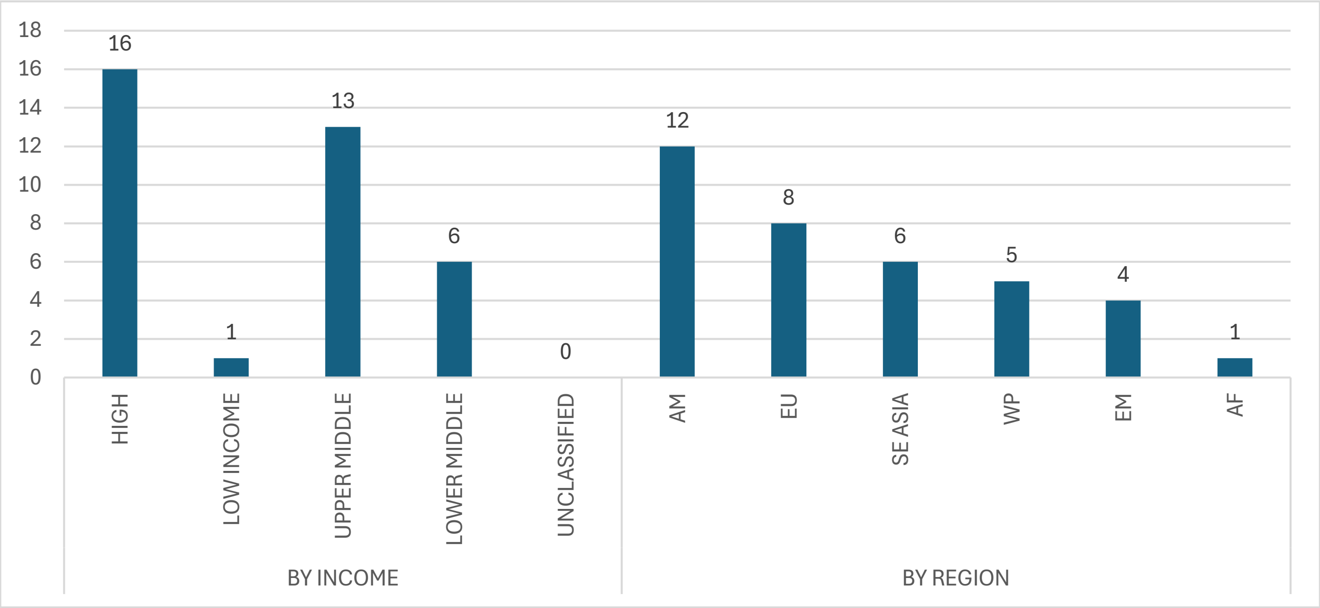

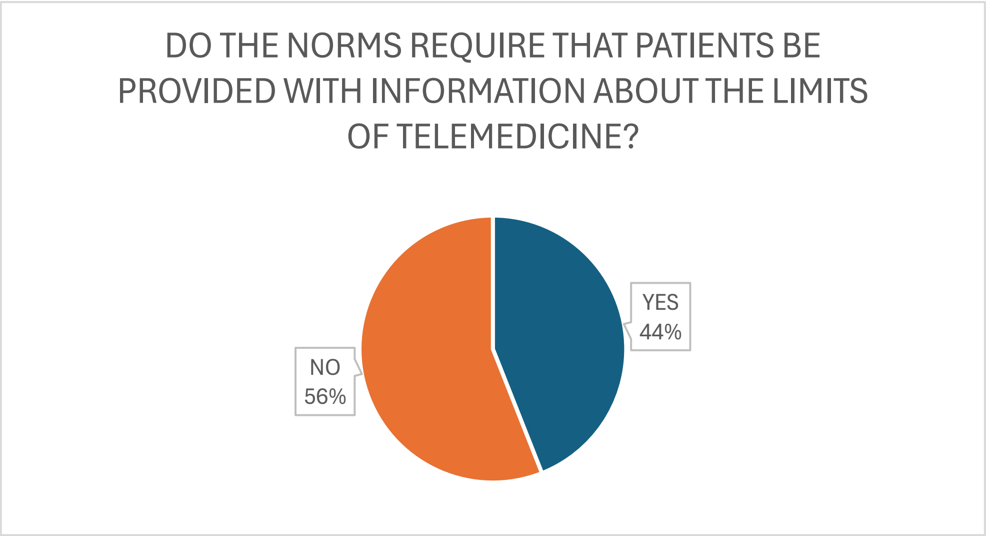

DO THE NORMS REQUIRE A PRIOR IN-PERSON CONSULTATION?

	AM	EU	SE ASIA	WP	EM	AF	WORLD	PROPORTION		
HIGH	0	3	0	0	0	0	3	4%	YES	10%
LOW INCOME	0	0	0	0	0	1	1	1%	NO	90%
UPPER MIDDLE	1	1	1	0	0	0	3	4%		
LOWER MIDDLE	0	0	0	1	0	0	1	1%		
UNCLASSIFIED	0	0	0	0	0	0	0	0%		
	1	4	1	1	0	1	8	10%		

BY INCOME					BY REGION					
HIGH	LOW INC	UPPER MI	LOWER M	UNCLASS	AM	EU	SE ASIA	WP	EM	AF
3	1	3	1	0	1	4	1	1	0	1

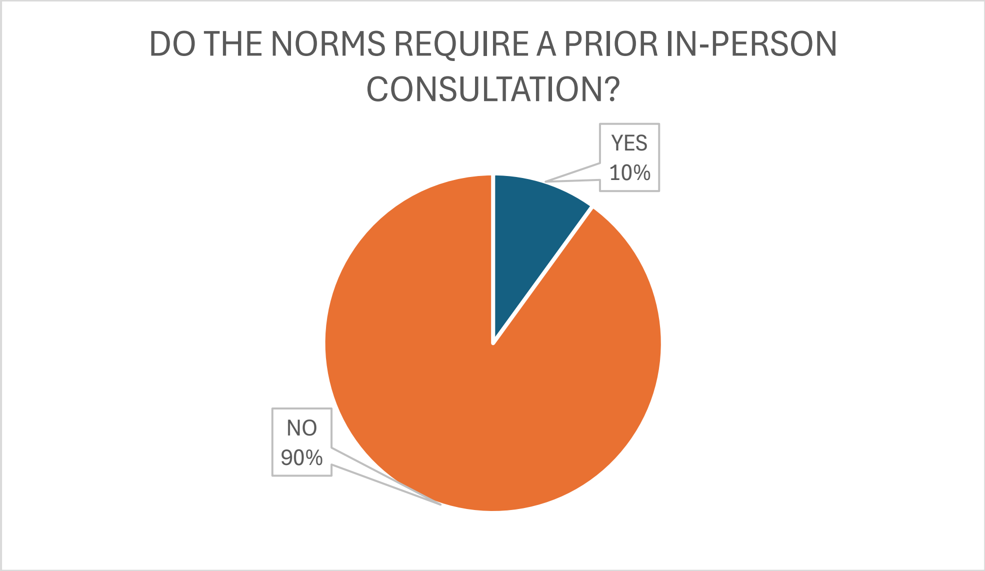

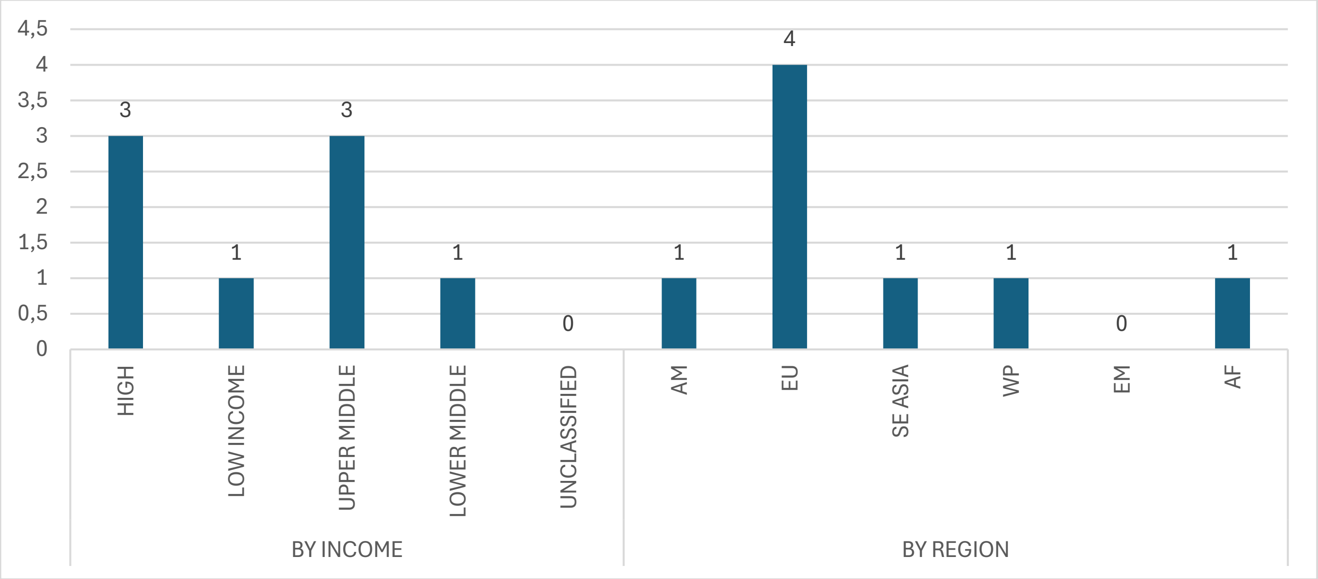

IS THERE A MONITORING MECHANISM IN PLACE?

	AM	EU	SE ASIA	WP	EM	AF	WORLD	PROPORTION		
HIGH	4	17	0	4	4	0	29	36%	YES	80%
LOW INCOME	0	0	0	0	0	3	3	4%	NO	20%
UPPER MIDDLE	12	3	3	1	0	1	20	25%		
LOWER MIDDLE	1	1	3	2	4	1	12	15%		
UNCLASSIFIED	1	0	0	0	0	0	1	1%		
TOTAL	18	21	6	7	8	5	65	80%		

BY INCOME					BY REGION					
HIGH	LOW INC	UPPER M	LOWER M	UNCLASS	AM	EU	SE ASIA	WP	EM	AF
29	3	20	12	1	18	21	6	7	8	5

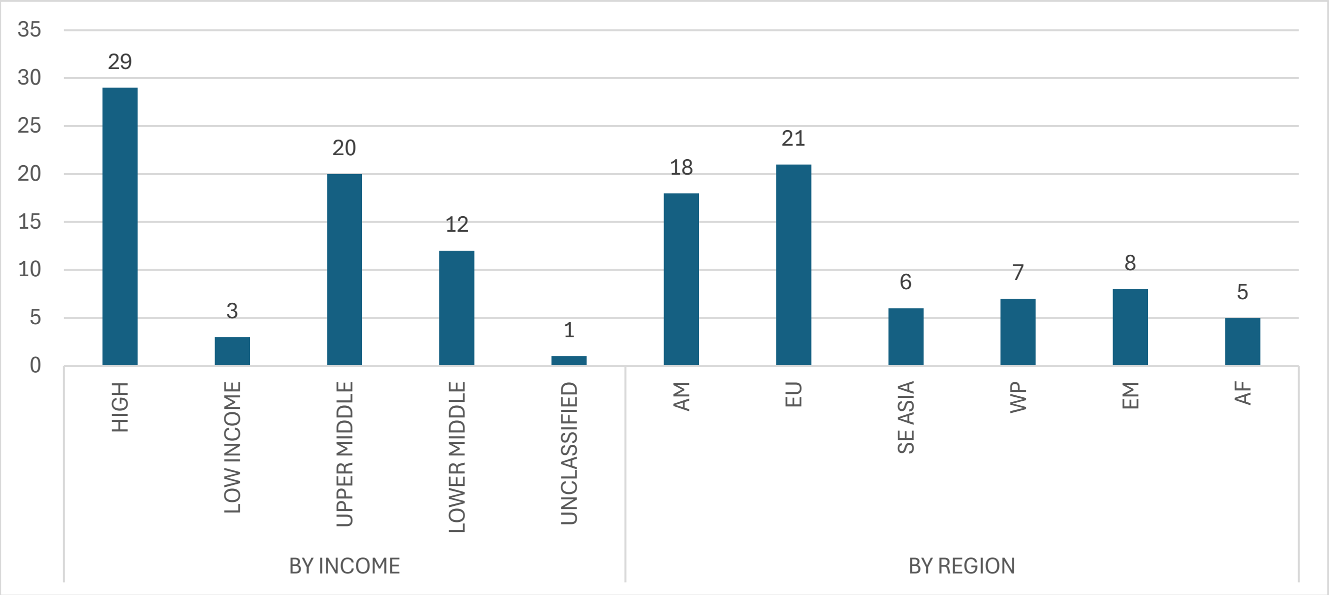

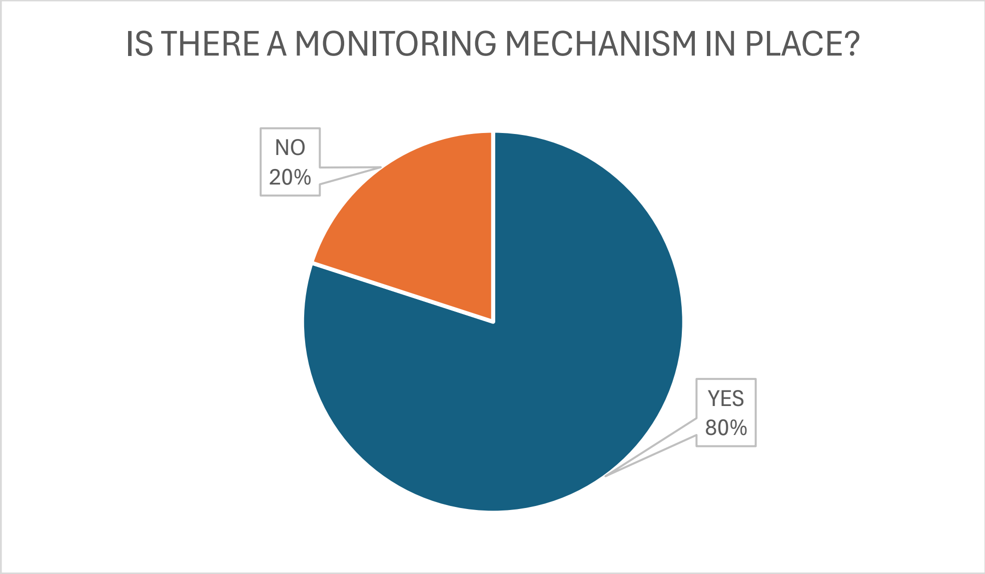

IS IT NECESSARY TO HAVE ANY TRAINING IN TELEMEDICINE TO PROVIDE CARE IN THIS MODALITY?

	AM	EU	SE ASIA	WP	EM	AF	WORLD	PROPORTION		
HIGH	2	4	0	2	4	0	12	15%	YES	32%
LOW INCOME	0	0	0	0	0	0	0	0%	NO	68%
UPPER MIDDLE	8	0	1	0	0	0	9	11%		
LOWER MIDDLE	0	0	3	1	1	0	5	6%		
UNCLASSIFIED	0	0	0	0	0	0	0	0%		
TOTAL	10	4	4	3	5	0	26	32%		

BY INCOME					BY REGION					
HIGH	LOW INC	UPPER MI	LOWER M	UNCLASS	AM	EU	SE ASIA	WP	EM	AF
12	0	9	5	0	10	4	4	3	5	0

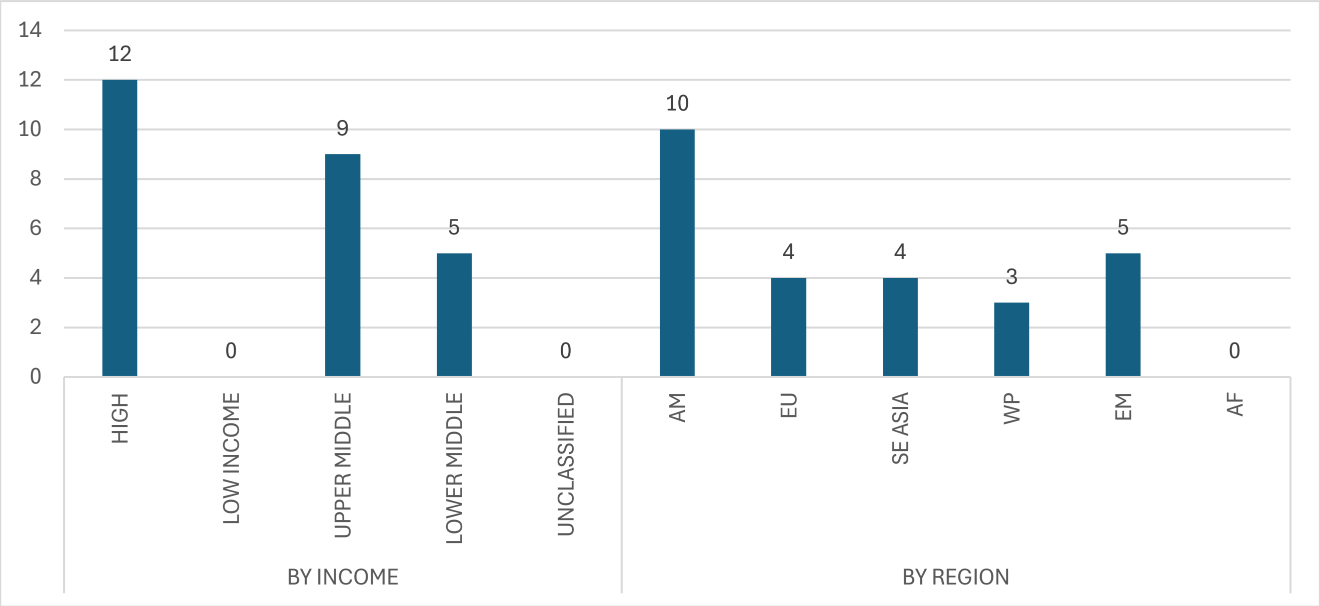

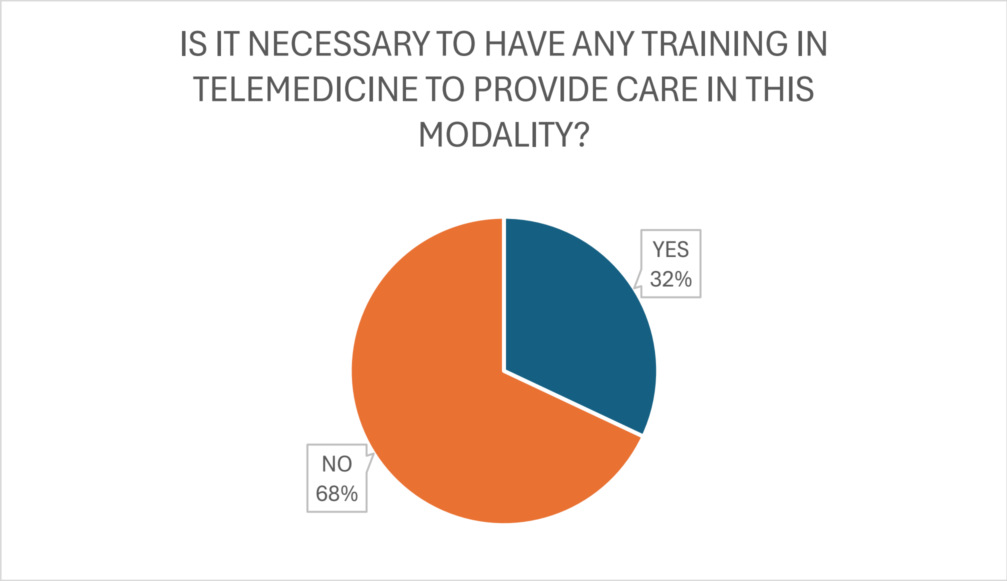

DO THE NORMS MENTION THE PRINCIPLE OF JUSTICE IN ANY WAY?

	AM	EU	SE ASIA	WP	EM	AF	WORLD	PROPORTION		
HIGH	3	10	0	3	1	0	17	21%	YES	52%
LOW INCOME	0	0	0	0	0	1	1	1%	NO	48%
UPPER MIDDLE	9	0	3	0	0	1	13	16%		
LOWER MIDDLE	1	2	3	1	3	0	10	12%		
UNCLASSIFIED	1	0	0	0	0	0	1	1%		
TOTAL	14	12	6	4	4	2	42	52%		

BY INCOME					BY REGION					
HIGH	LOW INC	UPPER MI	LOWER M	UNCLASS	AM	EU	SE ASIA	WP	EM	AF
17	1	13	10	1	14	12	6	4	4	2

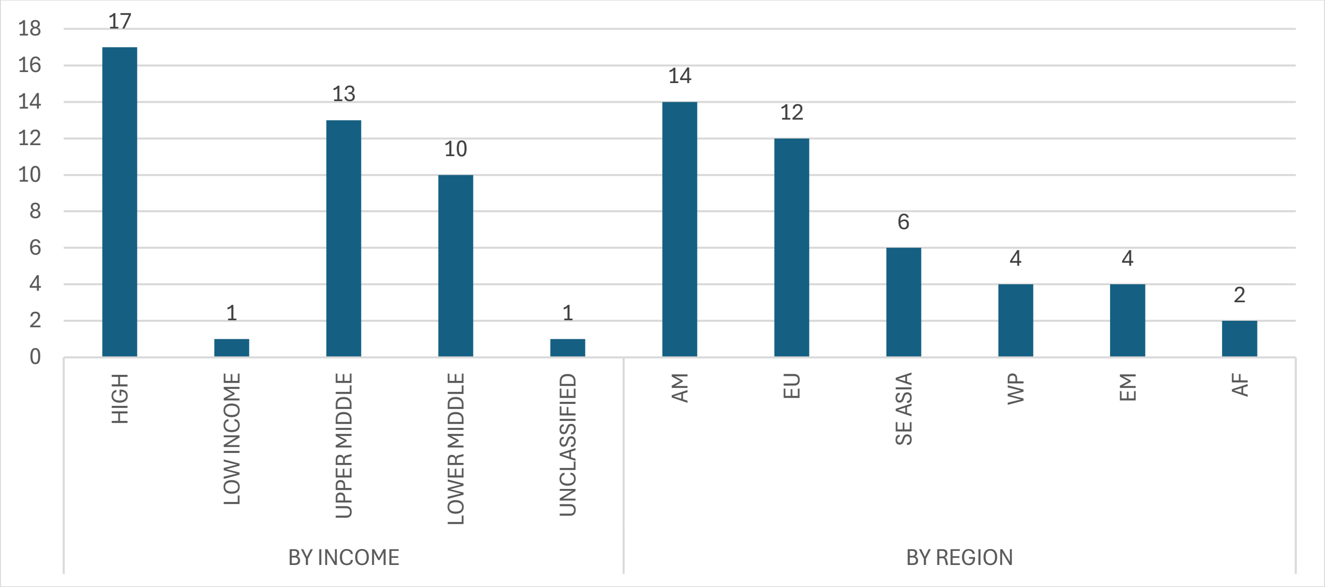

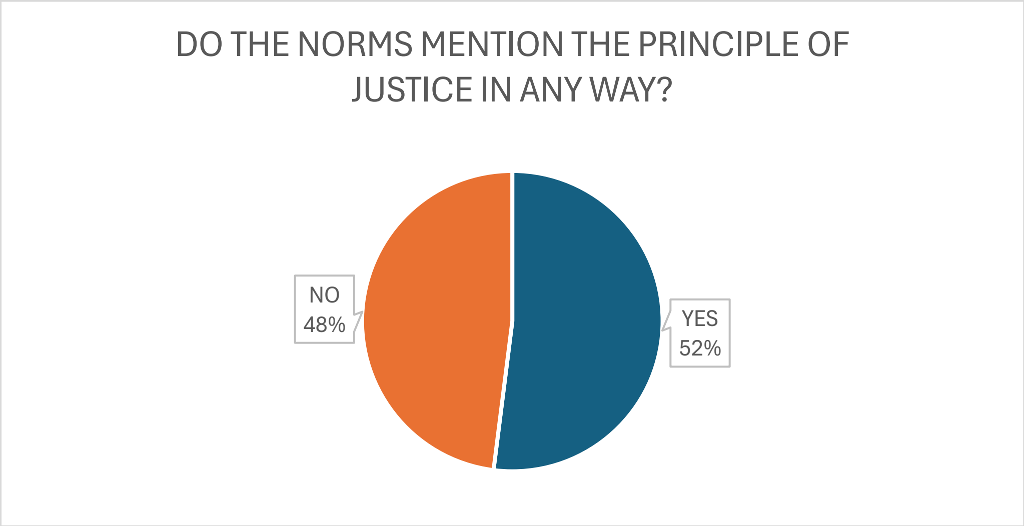

IS THERE A GUARANTEE FOR REDUCING ACCESS BARRIERS?

	AM	EU	SE ASIA	WP	EM	AF	WORLD	PROPORTION		
HIGH	3	2	0	0	2	0	7	9%	YES	31%
LOW INCOME	0	0	0	0	0	2	2	2%	NO	69%
UPPER MIDDLE	7	2	2	0	0	0	11	14%		
LOWER MIDDLE	0	0	2	0	3	0	5	6%		
UNCLASSIFIED	0	0	0	0	0	0	0	0%		
TOTAL	10	4	4	0	5	2	25	31%		

BY INCOME					BY REGION					
HIGH	LOW INC	UPPER MI	LOWER M	UNCLASS	AM	EU	SE ASIA	WP	EM	AF
7	2	11	5	0	10	4	4	0	5	2

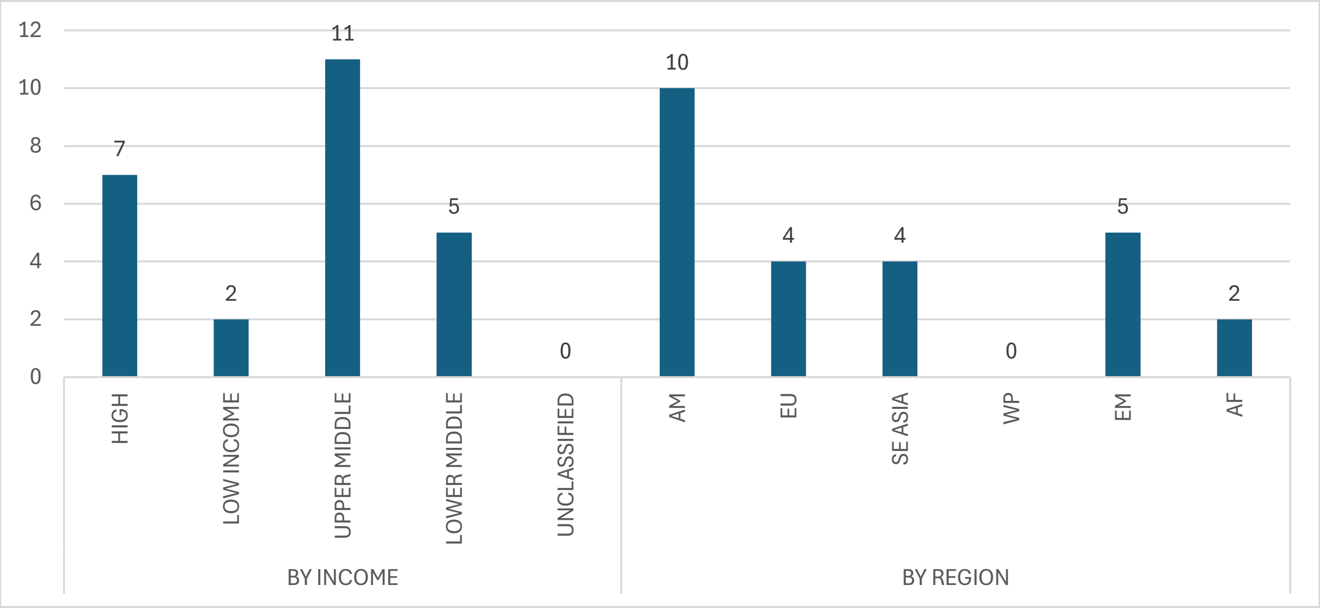

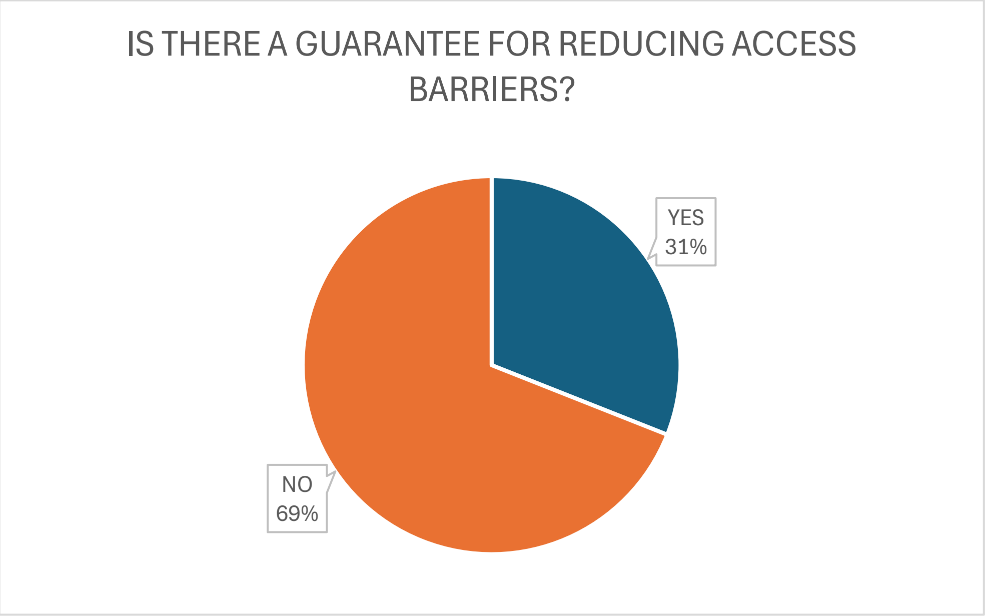

Supplement: Multimedia Appendix 2 [file formative-v10-e86613-s002.pdf]
